# Microglia–Astrocyte Interaction in Neural Development and Neural Pathogenesis

**DOI:** 10.3390/cells12151942

**Published:** 2023-07-27

**Authors:** Meiqi Sun, Hongli You, Xiaoxuan Hu, Yujia Luo, Zixuan Zhang, Yiqun Song, Jing An, Haixia Lu

**Affiliations:** 1 Department/Institute of Neurobiology, School of Basic Medical Science, Xi’an Jiaotong University Health Science Center, Xi’an 710061, China; smqmina44@163.com (M.S.); youhongli2002@163.com (H.Y.); xiaoxuanhu21@163.com (X.H.); lyj13708151072@163.com (Y.L.); zhangzixuan_dsa@163.com (Z.Z.); syq2836878839@stu.xjtu.edu.cn (Y.S.); 2 Department of Human Anatomy & Histoembryology, School of Basic Medical Sciences, Xi’an Jiaotong University Health Science Center, Xi’an 710061, China

**Keywords:** microglia–astrocyte interaction, neuroinflammation, neurodevelopment, astrogliogenesis, neurogenesis, neural pathogenesis

## Abstract

The interaction between microglia and astrocytes exhibits a relatively balanced state in order to maintain homeostasis in the healthy central nervous system (CNS). Disease stimuli alter microglia–astrocyte interaction patterns and elicit cell-type-specific responses, resulting in their contribution to various pathological processes. Here, we review the similarities and differences in the activation modes between microglia and astrocytes in various scenarios, encompassing different stages of neural development and a wide range of neural disorders. The aim is to provide a comprehensive understanding of their roles in neural development and regeneration and guiding new strategies for restoring CNS homeostasis.

## 1. Introduction

In the central nervous system (CNS), there are a variety of glial cells including astrocytes, oligodendrocytes, and microglia [1]. Astrocytes are the most abundant type of glial cells and play numerous roles in brain function. Oligodendrocytes form the myelin sheath of neuronal axons, which is required for the proper conduction of neuronal signaling. Microglia are known as the monocytes of the CNS and specialize in the phagocytosis of pathogens and debris although they are considered as glial cells.

Astrocytes are of ectodermal origin while microglia originate from the yolk sac of an embryo [2,3]. Despite originating from diverse sources, they exhibit close proximity in their anatomical locations within the CNS. Microglia are uniformly distributed and perform conserved functions throughout the brains of various species [4,5], enabling them to actively and efficiently survey their environments. Astrocytes display heterogeneity in distinct brain regions [6,7], maintaining close contact with synapses and microglia [8], thereby acting as links for the collective functionality of neural networks. Microglia and astrocytes do not solely function as supporting cells of neurons. It has been demonstrated that they play dual roles during development and neural regeneration [9]. Under normal physiological conditions, they remain in resting states. Upon bodily stimulation or during disease progression, both astrocytes and microglia transition into reactive states, yielding a double-edged effect. In this study, we use the terms “active” and “reactive” to characterize astrocytes and microglia, replacing the use of A1/A2 astrocyte classifications [10] and M1/M2 microglia [11], because the latter kind of classification method cannot sufficiently differentiate functional phenotypes based on single-cell sequencing data [12]. In line with this, microglia–astrocyte interactions change during such periods. The importance of the microglia–astrocyte interaction in the neural development and progression of diseases should not be underestimated. These interactions guide neuron generation and maturation, the clearance of cellular debris, and the maintenance of homeostasis in the CNS during development. During diseases, they become activated and display both similar and distinct functions, supporting and regulating each other. Therefore, there has been a recent surge of interest in the interaction between astrocytes and microglia, including their specific anatomical locations and functions in different scenarios [13,14].

The activation of astrocytes and microglia after injury has been previously discussed extensively. However, the importance of the “glial cell niche”, which is composed of different types and originates from glial cells, has not received sufficient attention. Throughout neural development, microglia–astrocyte interactions provide important clues for neural stem/progenitor cells and help maintain homeostatic balance by means of morphological alteration and via molecular interactions. From normal physiological states to abnormal states, when the balance is disrupted, the transition from the resting and active modes of their interaction to a reactive mode becomes crucial for regulating inflammation and regeneration. Due to the variability and complexity of their interactions, it is necessary to comprehensively review their roles in every stage of development and in various brain disorders. This paper aims to examine the similarities and differences in activation modes between microglia and astrocytes in different scenarios, including nearly all stages of neural development, and in various neurological disorders such as infectious inflammation, trauma, and degenerative disorders.

## 2. Microglia–Astrocyte Interaction during Neural Development

During rodent embryonic development, neuroepithelial cells transform into radial glial cells, which undergo a transition from unipotent intermediate progenitor cells to pluripotent intermediate progenitor cells. The former generate pyramidal neurons and the latter produce astrocytes during late embryonic development (embryonic days E17.5) [15]. The molecules and various influencing factors that are related to the switch from neurogenesis to gliogenesis are crucial for specific brain cell architecture and functional homeostasis. Microglia function as extracellular factors that affect the transition from neurogenesis to gliogenesis [16].

### 2.1. Microglia and Neurogenesis

**Figure 1:**
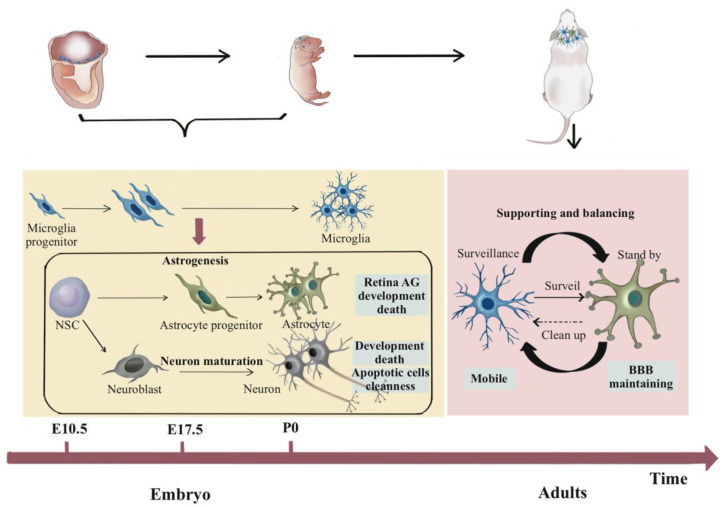
Microglia–astrocyte interactions during neurodevelopment and under physiological conditions. Microglia are derived from the yolk sac and colonize the central nervous system in the early embryonic period, guiding neurogenesis and the distribution of neurons. Additionally, they act as key cellular factors in the late embryonic and early postnatal stages to promote astrogliogenesis. In adulthood, microglia normally scan surrounding cells, while astrocytes express Tyro3, Axl, and MerTK receptors on standby. Astrocytes will change their responses when microglia are damaged. CNS: central nervous system; BBB: blood–brain barrier; NSC: neural stem cell.

Microglia, exhibiting amoeboid morphology, migrate to the CNS prior to the onset of neurogenesis [17]. They can be observed in the brain rudiment as early as E8 [18] and colonize the CNS parenchyma by E10.5 (Figure 1). Upon maturation and radial spreading throughout the brain, fully branched microglia with distinct morphological characteristics emerge on E28 [19,20]. It has been confirmed that the development and maturation of microglia are synchronized with neurogenesis.

The first group of neurons are generated on E10 [21,22] and the neurogenesis continues from E11.5 to E18.5 [23]. Microglia directly interact with neurons through surface molecules and mediate developmental neuron death [24,25] (Figure 1). These microglia-mediated neuron deaths also provide the conditions for both the subsequent astrogliogenesis as well as the astrocyte maturation and precise distribution. In addition, the interaction between microglia and astrocytes has a significant influence on neural maturation. Specifically, the phagocytic abilities of microglia and astrocytes have a strong effect on synaptic pruning. During neuronal corpse removal, small dendritic apoptotic bodies are engulfed by astrocytes, and the soma and apical dendrites are engulfed by migrated microglia [26]. In addition, IL-33 secreted by developing astrocytes increases the phagocytic ability of microglia, and the insulin-like growth factor 1 (IGF-1) secreted by microglia enhances the astrocytes’ phagocytic capacity. They both further accelerate neuron maturation [27,28]. Besides synaptic pruning, the interaction between microglia and astrocytes contributes to the balancing of synaptic number and structure/function.

### 2.2. Microglia and Astrogliogenesis

Studies have been conducted both in vivo and in vitro to investigate the effect of microglia on gliogenesis. An in vivo study demonstrated that ventricular-residing microglia acquire an activated phenotype and regulate gliogenesis during development. Microglia influence the development of tuberal hypothalamic glial populations. Microglia facilitate the phenotype of PdgfRα^+^ oligodendrocyte progenitor cells (OPCs) and Olig2^+^ progenitors but do not affect astrogliogenesis [29]. In contrast, an in vitro study using microglia-deficient mice showed that the neuronal generation capacity did not change but the number of new generated astrocytes decreased. The addition of microglia could rescue astrocyte shortages [30]. This study indicated that microglia facilitate the generation of astrocytes in vitro. Further study confirmed that the soluble factors secreted by microglia, including interleukin-6 (IL-6) and leukaemia inhibitory factor (LIF), and also the JAK/STAT and MAPK signaling pathways, were involved [31]. In addition to astrocyte generation, microglia also play a role in the developmental death of astrocytes. It has been shown that the death of retinal astrocytes during development is the result of microglia exerting a phagocyte-mediated death mechanism. A small number of astrocytes were killed by microglia during migration before postnatal day 5 (P5, Figure 1) while more than three times as many astrocytes were killed between P5 and P14. The death of astrocytes was significantly inhibited when microglia were ablated [32].

In adulthood, astrocytes and microglia share similar properties and functions in maintaining normal physiological functions, such as by supporting neurotransmission, energy conduction, and metabolism [33,34,35], balancing cell numbers and ratios [28], maintaining cellular heterogeneity [35,36], and carrying out phagocytosis (Figure 1). They also have unique characteristics on specific occasions. For example, most astrocytes are stationary and positionally stable in their quiescent state. They regulate cerebral blood flow by constituting and maintaining the blood–brain barrier (BBB) [35]. On the contrary, microglia are highly mobile and dynamic. In vivo two-photon imaging revealed that microglia of adult mice constantly patrol the microenvironment through movement and process retraction [37,38,39], even in the resting state. This allows microglia to rapidly respond to the changes of surrounding environments and to extensively repopulate after depletion [28].

Astrocytes are in a standby state under normal conditions. They can become compensatory when microglia are damaged. Existing data have also demonstrated that the targeted depletion of microglia, whether in *Siglech* ^dtr^ mice [40] or by utilizing PLX5622, results in the impairment of their phagocytic activity [41]. Consequently, the activation of astrocytes ensues, accompanied by an upregulated expression of pro-inflammatory genes. Activated astrocytes function in engulfing microglia fragments through mechanisms involving Tyro3, Axl, and MerTK (TAM) phagocytosis receptors and C4b-facilitated phagocytosis. This function is unique to astrocytes, and is not exhibited by other phagocytes (such as macrophages and monocytes) and non-phagocytic cells (such as endothelial cells, OPCs, oligodendrocytes, NSCs, neurons, and circulating blood cells).

## 3. Microglia–Astrocyte Interaction after Neural Injury

It is notable that under normal physiological conditions, a state of biological homeostasis exists within the microglia–astrocyte interaction. However, when the organism encounters disease, significant changes occur in the interaction as a result of inherent sensitivity characteristics of cells to stimuli. Damage-associated molecular patterns (DAMPs) [42] released by reactive cells play important roles in the neuroinflammation of neural disorders including traumatic brain injury (TBI) [43], stroke, or neurodegenerative diseases. Inflammatory responses are commonly accompanied by the activations of astrocytes and microglia. The sensitivities and responses of microglia and astrocytes change with various DAMPs.

### 3.1. Traumatic Injury

Traumatic injuries in the CNS include brain and spinal cord injuries. The interruptions of brain and spinal cord function are caused by external physical forces. The basic pathological changes include the external compression of mass, contusion, diffuse axonal injury, ischemia, apoptosis, mitochondrial dysfunction, cortical diffusion inhibition, microvascular thrombosis [44], toxic-chemical-releasing, and electrolyte shifts [45]. Symptoms in an acute period are exacerbated by episodes of chronic inflammation over time, at which point the activation of astrocytes and microglia is no longer neuroprotective and is often neurotoxic. Taking TBI as an example, damaged tissue or innate immune cells first respond to the injury and then recruit glial cells and T cells to the injury site to form a glial scar that surrounds and isolates the damaged tissue [46]. Chemokines and cytokines secreted by immune cells further enhance inflammation. Resident cells in the brain, such as microglia, astrocytes, and neurons [43], cooperate with immune cells to regulate the inflammation and prevent further tissue loss [47].

**Figure 2:**
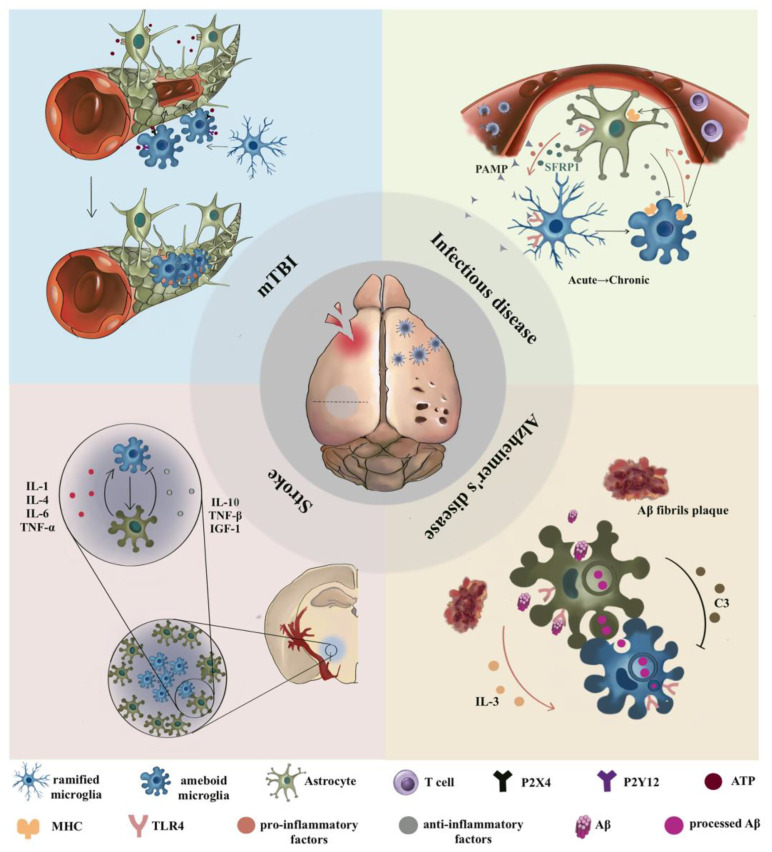
Microglia–astrocyte interactions during neuroregeneration and under disease states. Astrocytes and microglia show different interaction behaviors in diverse diseases. In mTBI, ATP synthesized by astrocytes binds to the upregulated P2X4 and P2Y12 receptors of microglia to promote their deformation and migration, eventually replacing the damaged astrocyte barrier. Patrolling microglia are activated when haemorrhagic stroke happens. They move to the lesion’s core and release inflammatory factors and anti-inflammatory factors. The former leads to the proliferation and recruitment of dendritic cells, T cells in the lesion, and an inflammatory storm, while the purpose of the latter is to inhibit microglia activation and promote lesion repair. In infectious diseases, Toll-like receptor 4 (TLR4) on astrocytes and microglia recognizes pathogen-associated molecular patterns (PAMPs) and then presents antigens to T cells. In neurodegenerative diseases, such as Alzheimer’s disease, both astrocytes and microglia can recognize amyloid-β (Aβ), and astrocytes act as primary processors, internalizing protein aggregates and performing primary processing. Intermediate products will either stay in astrocytes or be secreted and engulfed—and eventually cleared—by microglia. DAMPs: damage-associated molecular patterns.

Microglia–astrocyte interaction plays dual roles after traumatic injury. Secondary injury after TBI is the main cause of neurocognitive loss. During this period, damaged neurons, astrocytes, or cellular debris act as DAMPs to activate microglia within minutes after injury [43]. In mild TBI, microglia transform into highly mobile jellyfish-like phagocytes and perform neuroprotective roles. They insert themselves into the damaged glial junction in order to repair the structural damage of astrocytes or directly replace the dead astrocytes and connect together to form a phagocytic barrier (Figure 2). Along with this, microglia further clear the astrocyte debris from the injured site. The response of microglia has been confirmed to rely on purinergic receptor signaling (P2X4 and P2Y12) and the release of astrocyte-dependent adenosine triphosphate (ATP) [44,48]. In addition, astrocytes and microglia further cooperate with immune cells [43] and act as inflammatory amplifiers. Moreover, soluble mediators, such as reactive oxygen species and purines, trigger an inflammatory cascade and allow myeloid monocytes to enter the damaged brain, resulting in neurotoxicity [48].

Activated microglia recruited to the injury release the stromal cell-derived factor 1 (SDF1). It will bind with the C-X-C chemokine receptor type 4 (CXCR4) on astrocytes and lead to the production of TNFα, which potentiates the excitatory synaptic scaling mechanism [49]. In addition, microglia and astrocytes behave differently in terms of transcriptional signatures in mild TBI and in severe TBI. In severe TBI, transcriptional changes in microglia are greater than those in astrocytes. Both microglia and astrocytes have shown enrichment for genes related to type I interferon signaling. This is different from what is found in other CNS diseases [50].

### 3.2. Ischemic and Hemorrhagic Injury

Stroke is an ischemic lesion caused by the occlusion of cerebral arteries or, less frequently, a cerebral hemorrhage in the parenchyma or subarachnoid space. The mortality rate of hemorrhagic stroke is extremely high, reaching 80%. In the acute phase, innate immune cells invade and damage the brain and meninges [51]. Pro-inflammatory cytokines (IL-1β, IL-1α, and TNF-α), chemokines (CCL5, CXCL4, and CXCL7), and proteases (MMP8, MMP9, and MT6-MMP) are released by endothelial cells, perivascular macrophages, platelets, and neutrophils. Activated microglia also secrete IL-1β, IL-6, and TNF-α. These molecules play pivotal roles in initiating an inflammatory cascade by activating and recruiting nearby astrocytes, dendritic cells, and T cells [52].

Microglia–astrocyte interaction also plays dual roles after stroke. In ischemic stroke, the reactive astrocytes function as phagocytes within the ischemic penumbra region during the later stage of ischemia, while phagocytic microglia are mainly present in the ischemic core region in the early stages [53]. Microglia initially respond to DAMPs and then induce the activation of astrocytes [54]. The fragmented astrocytes act as important components of DAMPs and induce microglia activation in TBI. While in stroke, microglia act as investigators, and the blood-borne stimulation is the main DAMP. Activated microglia release cytokines, induce astrocyte activation via binding to glycoprotein 130 in astrocytes, and activate the JAK/STAT3 pathway. Microglia also regulate anti-inflammatory properties in astrocytes. Data from an in vitro experiment demonstrated that the capability of astrocytes to produce inflammatory mediators, including IL-1α, nitric oxide synthase (iNOS) and TNF-α, was significantly reduced when co-cultured with microglia. However, when microglia were ablated in the MCAO animal model, the production of IL-1α, IL-1β, inducible iNOS, TNF-α, and IL-6 by astrocytes was upregulated [55].

Among the activated astrocytes, the pro-inflammatory phenotype further promotes the inflammatory activation of microglia while the anti-inflammatory phenotype inhibits the action of microglia. Activated astrocytes produce a large number of inflammatory factors, including IL-1, IL-4, IL-6 and TNF-α, which induce excitotoxicity and an excessive inflammatory response. This has a negative effect on the recovery of stroke patients. They also produce IL-10, transforming growth factor beta (TGF-β), and IGF-1 to promote the resolution of inflammation and tissue repair [56] by relieving oxidative stress [57], protecting neurons, reducing brain edema, and decreasing the infarct volume (Figure 2).

### 3.3. Neuroinfections

#### 3.3.1. Pathogen-Associated Molecular Pattern Invasion

Compared to infections in other organs, CNS infections have higher morbidity and mortality. CNS infectious diseases are caused by various organisms. Examples of CNS infectious diseases include acquired immunodeficiency syndrome, rabies, Japanese encephalitis, and herpes simplex encephalitis, all caused by viruses; tuberculosis, syphilis, bacterial meningitis, and septicaemia, all caused by bacteria; cryptococcal meningitis, caused by fungus; and malaria, neurocysticercosis, and neuroschistosomiasis, all caused by parasitic infections [58,59]. The infection-associated molecular patterns, also known as pathogen-associated molecular patterns (PAMPs) or “stranger” signals, mainly come from microbes [60] or viruses [61]. The function of PAMPs is similar to that of the aforementioned DAMPs [42,62]. Furthermore, in infectious inflammation, PAMPs and DAMPs work together to trigger glial responses. The apoptotic debris and cell secretions triggered by the PAMPs act as DAMPs to further aggravate or regulate inflammatory responses.

#### 3.3.2. Roles of Microglia–Astrocyte Interaction in Infectious Inflammation

When PAMPs invade the brain, resident astrocytes and microglia [63] act as antigen-presenting cells. They both express major histocompatibility complex (MHC) molecules and costimulatory factors, performing the function of antigen presentation. Reactive astrocytes express MHC II and costimulatory molecules such as CD80 (B7-1) and CD86 (B7-2), which promote T cell activation. Microglia are thought to be the first cells to respond during infections because they mainly express TLRs and produce pro-inflammatory mediators in response to TLR ligands. In systemic infections, even when the BBB is intact, microglia are activated by circulating pathogens attacking circumventricular organs [64], and secrete a series of DAMPs that act on astrocytes. Furthermore, microglia respond to viral PAMPs, initially by limiting the first phase of viral replication through the inflammatory response and subsequently inhibiting this response to avoid severe neuronal damage [65] (Figure 2). Microglia could also be activated by metabolites of dietary tryptophan produced by the commensal flora, then release TGFα and vascular endothelial growth factor B (VEGF-B) to regulate the pathogenic activities of astrocytes in the experimental autoimmune encephalomyelitis (EAE) mouse model of multiple sclerosis [66].

In addition, activated astrocytes act on microglia to promote the transition from acute to chronic inflammation or maintain the progress of chronic inflammation. In mouse models of lipopolysaccharide (LPS)-induced acute and chronic neuroinflammation, astrocyte-derived secreted frizzled-related protein 1 promoted microglia activation and enhanced their response to injury, maintaining the state of chronic inflammation. It also upregulated components of the hypoxia inducible factor-dependent inflammation pathway and NF-kB [67], acting as an important intermediate molecule in the transition from acute to chronic inflammation.

## 4. Microglia–Astrocyte Interaction in Neurodegenerative Diseases

### 4.1. Protein Aggregates and Chronic Inflammation

Neurodegenerative diseases are characterized by progressive declines in motor and/or cognitive function caused by selective losses of neurons in the CNS [68]. Basic pathological changes are classified as amyloidosis, tauopathies, alpha-synucleinopathies, and transactivation-responsive DNA-binding protein 43 (TDP-43) proteinopathies. Abnormal protein conformations and their neuroanatomical distribution in these diseases are also histopathological features necessary for diagnosis. Abnormal protein degeneration and accumulation, like tauopathies, have been observed in neurons, astrocytes, and oligodendrocytes [69].

Chronic inflammation and the inadequate degradation of abnormal protein aggregates are the driving forces of brain pathology in Alzheimer’s disease (AD) and Parkinson’s disease (PD) [70]. AD is characterized by the extracellular accumulation of plaques containing amyloid-β (Aβ) and the intracellular neurofibrillary tangles induced by hyperphosphorylated tau protein [71]. Neuroinflammation occurs early in AD and contributes to the progression of the disease. PD is characterized by the accumulation of Lewy bodies in the substantia nigra, pars compacta, and other structures, intracellular inclusions containing alpha-synuclein (αSYN), and the deaths of dopaminergic neurons. It has been shown that neuroinflammation is involved in PD progression.

### 4.2. Dual Roles of Microglia–Astrocyte Interaction in Phagocytosis in Neurodegenerative Disorders

Significant changes in the morphologies of microglia and astrocytes occur in physiological aging and neurodegenerative diseases. In AD, the hypertrophy of astrocytes is characterized by an enlarged cell body and an increase in glial fibrillary acidic protein (GFAP), which is mainly observed in the core, corpus callosum, and hippocampus. Additionally, a decrease in the complexity of astrocytic processes has been observed in aging [72,73,74]. Microglia in AD also undergo morphological changes including increases in the sizes of the somas and the retraction of their processes [75]. The retraction of processes by both types of glial cells disrupts synaptic connections and compromises neural function. [76,77]. Moreover, aged astrocytes impair the efficiency of synaptic transmission [78] while microglia in aging exhibit reduced phagocytic activity [79,80], and both increase the release of pro-inflammatory factors [81]. These changes in morphology and cellular function serve as important links between aging and neurodegenerative processes.

In addition to morphological changes, the functions of astrocytes and microglia in AD and PD are also similar. They can bind to Aβ or αSYN and then activate downstream signaling pathways. Protein aggregates will accumulate significantly in the case of microglia and astrocyte metabolic dysfunction [14,82] during the progression of a disease. Microglia–astrocyte interaction affects the clearance capacity of their Aβ or αSYN aggregates. In the co-culture system of astrocytes and microglia, fewer intracellular deposits of αSYN and Aβ have been found than that in either single-type glial cell culture system.

Glial cell interaction in neurodegenerative diseases is triggered by the binding of DAMPs either to the Toll-like receptors (TLRs) on the cell membranes of microglia and astrocytes or to the intracellular receptors through endocytosis, resulting in downstream signaling activation [83]. Within this interaction, microglia and astrocytes may both act as the first sensors of DAMPs but with different behaviors due to different signaling pathways activated, factors secreted, and phagocytic functions even when they receive the same DAMP. In response to αSYN, TLR4 activation induces microglial phagocytic activity, pro-inflammatory cytokine release, and ROS production, subsequently triggering the pro-inflammatory transformation of astrocytes via the secretion of TNF-α, IL-1α, and C1q [84]. However, αSYN directly enters astrocytes through exocytosis and endocytosis, independent of the TLR4 pathway [84,85]. Furthermore, αSYN binding to FcγRIIB on microglia leads to the receptor-mediated downregulation of microglial phagocytosis, activating SHP-1 and further inhibiting phagocytosis [86].This is different from TBI and stroke, where microglia are activated first and release cytokines to activate astrocytes. It has been shown that microglia could attach to the cell membranes of astrocytes and then attract and remove intracellular protein deposits from astrocytes based on live-cell imaging data [70]. They may also convey signals to astrocytes and then foster anti-inflammatory activity or promote inflammation.

When they perform the clearance capacity of Aβ or αSYN aggregates, astrocytes play the role of a superficial processor. The protein deposit is completely decomposed and secreted by microglia. In one hand, astrocytes promote the ability of microglia to phagocytose by secreting cytokines like IL-3. IL-3 could trigger transcriptional, morphological, and functional programming in microglia, conferring an acute immune response program, and enhance their motility. Microglia accordingly express more IL-3Rα, the specific receptor for IL-3, after recognizing Aβ deposits. This then consequently alleviates AD pathology and cognitive decline [87]. On the other hand, astrocytes do not always promote the phagocytic capacity of microglia. It has been shown that Aβ could activate the nuclear factor kappa B (NF-κB) pathway in astrocytes and trigger the extracellular release of C3. Then, C3 will interact with the C3aR on neurons and microglia, leading to altered cognitive function and impaired Aβ phagocytosis and resulting in disease aggravation [88].

## 5. Discussion

Glial cell interaction exists in both neurogenesis and neuropathogenesis. Microglia colonize the CNS at an early stage of development and act as the key regulators in the transition from neurogenesis to astrogliogenesis. From then, microglia and astrocytes interact with each other routinely. Microglia are more active than astrocytes, even in a resting state. They perform similar functions such as helping synapse formation and pruning in some cases. They also aid or interfere with each other’s work sometimes. When astrocytes are damaged, microglia exhibit astrocyte-like function, and during TBI they replace the damaged astrocytes to maintain the integrity of the BBB. In some cases, astrocytes and microglia functionally complement each other in a mutually reinforcing manner or act as each other’s modulators. Although the molecular mechanisms underlying interactions may vary across different diseases, they can still be categorized into several major types. Moreover, these findings provide potential guidelines for the clinical management of inflammation.

### 5.1. Manifold Manifestations and Intricate Molecular Mechanisms Underlying Microglia–Astrocyte Interaction

The events within microglia–astrocyte interaction include direct contact, cytokine secretion, complement-mediated interaction, receptor regulation, and exocytosis. The ion channels and ATP-mediated Ca^2+^ conduction may be also involved [89]. Different disorders have their own emphases. Regarding the key issues of current research, receptors in TBI, cytokines in stroke, and complements in neurodegenerative diseases have been mainly studied.

Through direct contact, microglia phagocytose and scavenge fragments of astrocytes in normal conditions, and astrocytes phagocytose fragments of microglia when microglia are damaged. When it comes to indirect contact, microglia promote the secretion of anti-inflammatory factor IL-10 and Orosomucoid 2 (ORM2) [90] from astrocytes during chronic inflammation. This in turn inhibits cascade amplification, thereby restricting excessive inflammation. In some other cases, activated microglia induce astrocytic necroptosis through the TLR4/MyD88 signaling pathway [91]. The complement system also joins in the glial cell interactions, particularly in LPS-induced inflammation. Activated microglia further activate astrocytes by secreting C1q and cytokines. Activated astrocytes lose the ability to promote neuronal survival, growth, and synaptogenesis and gain the ability to induce neuronal and oligodendrocyte death [10]. Cytokines involved in the microglia–astrocyte interaction are extremely complicated due to their variety and the inflammatory storm. It is very difficult to identify the sources of cytokines. It is not easy to decipher the specific roles of individual factors either. Nonetheless, the diligent efforts of researchers have produced invaluable discoveries. As in the previous statement on TBI, IL-3 secreted by astrocytes acts on microglia, resulting in the recruitment of microglia to regulate tissue damage [92]. In another study, astrocytes secreted IL-10 in adult mice after facial nerve axonal microsurgery, altering the microglial activation, which is presented by phenotype, number, and density [93]. Receptors expressed in specific cells during inflammation were used to explain the complexity [94]. For example, cortical microglia can transform astrocytes into a neuroprotective phenotype by reducing P2Y1 purinergic receptor expression, promoting the formation of glial cell scars [95]. As for exocytosis, exosomes wrap RNA, proteins, lipids, or other contents that are included. Exosome-carried microRNAs, such as *miR-873a-5p* from activated astrocytes, could inhibit the NF-κB signaling pathway in microglia, leading to the attenuation of neuroinflammation and neurological deficits [96].

Neurons may be involved in microglia–astrocyte interaction as well. In most cases, neurons are taken as the downstream effectors. In an infection of the nervous system, ATP will be rapidly released by microglia and amplified by astrocytes as soon as they are activated. Then the upregulation of P2Y1 receptors in astrocytes increases excitatory postsynaptic current frequency [97]. It has also been shown that neurons might work as the intermediaries within glial cell interactions. During early postnatal periods, the expression of C1q in retinal ganglion cells is upregulated by activated astrocytes. This tags the removal of unwanted synapses by microglial phagocytosis via the classical complement pathway [98].

### 5.2. Future Research and Clinical Prospects

Overall, since the microglia play important roles in neurogenesis and gliogenesis, although they are not the original residents of the CNS, more attention should be given on the specific timing and pathways of microglia–astrocyte interaction and the underlying mechanisms in the hope of better understanding the precise regulation of neural development.

Neuroinflammation plays dual roles during neuroregeneration as mentioned above. The mixed pro- and anti-inflammatory effects occur in different spatial–temporal occasions in TBI, stroke, and neurodegeneration. Neuroinflammation in TBI is protective in the acute phase and harmful in the chronic phase. Chronic inflammation may develop into neurodegenerative diseases in the long term. While in stroke, the acute phase of the inflammation causes damage and inflammation, but in the terminal phase, it may have protective effects and lay the foundation for the post-ischemic repair process. Similarly, microglia and astrocytes in inflammatory responses act as pro- or anti-inflammatory agents that vary across the timeline. Microglia promote astrocyte pro-inflammatory effects and transition to a protective phenotype while astrocytes reciprocally regulate microglial characteristics via cytokines and exosomes [99]. However, the extent to which these interactions contribute to the ‘double-edged sword’ effect and the specific mechanisms involved need to be further investigated.

## 6. Conclusions

Microglia–astrocyte interaction would be the next key point in both basic research and translational study. Considering proper timing and implementing effective treatment according to the specific brain regions [100] should be considered instead of simply inhibiting the effects of cytokines or astrocytes and microglia. Studies on microglia and astrocyte surface proteomes and secretomes to understand the interactions in different situations are needed [101]. The precise regulation of the interactions between microglia and astrocytes leading to neuroprotective responses is required.

## Data Availability

Not applicable.
